# Strengthening Capacity for Tailored Immunization Programs Using Adult Learning Principles: A Case Study from Nigeria

**DOI:** 10.9745/GHSP-D-23-00465

**Published:** 2024-10-29

**Authors:** Chisom Obi-Jeff, Funmilayo Oguntimehin, Abduljaleel Adejumo, Abdulrahman Ibrahim, Olympus Ade-Banjo, Dan Gadzama, Nicholas Okoli, Chidera Obi, Rachael Olorupo, Ifeyinwa Martins, Amina Usman, Audu Joy, Tanko Chadwafwa, Anthony Onimisi

**Affiliations:** a Brooks Insights, Abuja, Federal Capital Territory, Nigeria.; b London School of Hygiene and Tropical Medicine, London, United Kingdom.; c Federal Capital Territory Primary Health Care Board, Abuja, Federal Capital Territory, Nigeria.

## Abstract

**Introduction:** Nigeria has the highest number of children who have not received any vaccines in Africa. The training-of-trainers (TOT) model used to train program managers (PMs) and health care workers (HCWs) is ineffective for adult learning and limits immunization programs’ success. We incorporated adult learning principles (ALPs) in designing and delivering TOT for immunization PMs and HCWs to use data to engage communities for tailored immunization strategies.

**Methods:** Our study was implemented in 3 local government areas (LGAs) of the Federal Capital Territory, Nigeria. A training curriculum was developed, integrating ALPs and technical and operational content based on best practices in delivering immunization training and the training needs assessment findings. State PMs (n=10), LGA PMs (n=30), and HCWs (n=42) were trained on the human-centered design for tailoring immunization programs (HCD-TIP) approaches using ALPs. We used interviews and surveys with purposively and conveniently sampled PMs and HCWs, respectively, and observations to assess participants’ satisfaction, knowledge and competence, behavior changes, and results. The interviews were analyzed thematically, and surveys were statistically.

**Results:** There was a high level of satisfaction with the training among LGA PMs (100%), state PMs (91%), and HCWs (85%), with significant knowledge and competence improvements post-training (*P*<.001). The trained participants conducted 2 HCD sessions with 24 undervaccinated communities and co-designed 24 prototype solutions for testing. Results showed increased coverage of the pentavalent vaccine first dose (54%) and third dose (188%) across 12 participating communities. Improved community colaboration, communication skills, and data-driven approaches were the most cited behavior changes in practice.

**Conclusion:** The application of ALPs in training, use of HCD-TIP approaches and tools, and supportive supervision enhanced PMs’ and HCWs’ capacity for tailored interventions. Countries should consider adopting a holistic approach that focuses on using these approaches in immunization programs to strengthen the health system for equitable vaccine coverage.

## Abstract (precis)

Incorporating participatory approaches such as adult learning principles in training PMs and HCWs improves their knowledge and skills in effectively engaging the communities and co-designing tailored interventions to improve vaccination uptake.

## Teaser

**Key Findings:**

- Skilled immunization program managers (PMs) and health care workers (HCWs) are critical for influencing vaccine uptake, which is essential for improving immunization coverage; however, the “training of trainers” (TOT) model used to deliver training neglects the different learnings needs of adults, resulting in reduced program effectiveness.
- Adult learning principles (ALPs) were used to design and deliver the TOT for PMs and HCWs on Human-Centered Design for Tailoring Immunization Programs (HCD-TIP).
- Three months post-training, data revealed increased vaccine uptake and behavior change in work practices, specifically increased PMs and HCWs’ capacity to engage undervaccinated communities to address barriers to vaccination and co-design people-centered, culturally appropriate, practical, and low-cost solutions to improve vaccination uptake and service delivery.

**Key Implications:**

- Program implementers should incorporate ALPs in designing and delivering capacity-building interventions for PMs and HCWs for an effective immunization program.
- Policymakers should create policies to integrate behavioral science into immunization programs to address the behavioral and social drivers of vaccine uptake and incorporate HCD approaches when engaging with communities to enhance people-centered, participatory, and data-driven interventions for equitable immunization coverage.

## INTRODUCTION

Vaccination is one of the most cost-effective and equitable public health interventions essential to primary health care (PHC), averting an estimated 4.4 million deaths annually.^1^ However, the underutilization of vaccines globally is concerning despite the ongoing recovery efforts from the COVID-19 pandemic. In 2022, 20.5 million children were either unvaccinated or undervaccinated, and 14.3 million did not receive any vaccines, referred to as zero-dose children.^2^ Unfortunately, under 60% of these children live in 10 countries, including Nigeria.^3^

Nigeria has one of the highest numbers of zero-dose children (2.2 million) in Africa and globally.^3^^–^^5^ This puts millions of children at risk of vaccine-preventable diseases and deaths. While reasons for non- or undervaccination are complex and multifaceted,^6^^,^^7^ interventions to increase childhood vaccination coverage are heavily skewed toward community engagement, sensitization, and mobilization activities, vaccination reminders, integration with other interventions, vaccination campaigns, and polio eradication efforts,^7^^–^^12^ with scholars calling for immunization program managers (PMs) and health care workers (HCWs) to develop tailored strategies to improve vaccine uptake, given their understanding of the context.^6^^,^^7^^,^^13^

However, there are competency gaps among immunization PMs and HCWs in Nigeria in data use and designing tailored immunization programs, contributing to suboptimal vaccine uptake and coverage.^8^^,^^14^^–^^18^ One key challenge is the knowledge and skill gap in basic immunization concepts, components of the Reaching Every Ward strategy, use of existing data to diagnose barriers to vaccination, and collaboration with communities to design and implement tailored interventions to improve vaccine coverage and equity in underserved communities.^14^^,^^17^^,^^19^ HCWs also lack communication skills, compromising their ability to effectively address vaccine hesitancy issues and deliver vaccination messages.^7^^,^^16^^,^^20^

Skilled immunization PMs and HCWs are critical for providing quality PHC services, including influencing vaccine uptake, which is essential for improving immunization coverage.^7^^,^^21^ The National Primary Health Care Development Agency (NPHCDA) conducts training for PMs and HCWs to deliver quality routine immunization (RI) services at the state and local government area (LGA) levels. The training is delivered through the cascade or “training of trainers” (TOT) model, where selected national PMs (master trainers) are trained and cascade the training to state PMs. These trained state PMs train the LGA PMs, who finally train the HCWs. While this model is effective, economical, and rapidly scalable, there have been concerns about the mode of delivery in TOTs with insufficient attention to the different learning needs of adults as it is transmitted at the lower levels,^22^^–^^24^ resulting in reduced learning outcomes and program effectiveness.^22^^,^^23^^,^^25^^–^^27^ These issues highlight the need to incorporate more effective, practice-oriented training approaches in the TOT model for the immunization workforce.

Applying the adult learning principles (ALPs) in the TOT model is recommended for public health training programs because it is a participatory, self-directed, problem-based, and experiential approach to learning.^24^^,^^28^^,^^29^ It recognizes that adults learn by actively participating in the learning process through discussions, problem-solving/brainstorming, and other interactive activities such as role-play and group exercise and can apply knowledge gained to real-life situations.^28^ However, the successful implementation of ALPs in TOT requires that the (1) training delivery approach is experiential and reflective at all levels, (2) training content is context-specific and involves a cross-section of stakeholders in preparing the training materials, and (3) training is followed up through mentorship and reporting.^24^^,^^25^^,^^28^

Evidence has shown that applying the best practices of ALPs in designing and delivering the TOT of an immunization program improved vaccination services and outcomes and can be used to design and implement high-quality training in resource-limited settings.^25^^,^^30^^,^^31^ Therefore, incorporating the best practices of ALPs could be used in designing and delivering training for immunization PMs and HCWs to use immunization data to diagnose barriers to vaccination and engage communities to co-design and implement tailored immunization programs to improve vaccine uptake and coverage.

Applying the best practices of ALPs in designing and delivering the TOT of an immunization program can be used to design and implement high-quality training in resource-limited settings.

Co-designing tailored immunization strategies is crucial for overcoming vaccine uptake and equity barriers.^32^^–^^40^ One approach to co-designing tailored vaccine strategies is the human-centered design (HCD) approach, which involves engaging communities to understand reasons for low uptake and co-designing viable and culturally appropriate solutions to address identified challenges.^37^^,^^39^^–^^42^ Using an HCD approach has increased vaccine uptake, community engagement, and local ownership in Nigeria^42^^,^^43^ and other low and middle-income countries (LMICs).^44^^–^^48^ To leverage these benefits, training PMs and HCWs on HCD approaches to enable them to effectively engage communities for tailored strategies is essential, and incorporating ALPs in designing and delivering the training can enhance this capacity-building process.

Using the latest evidence on vaccination behaviors and recognizing population diversity, the HCD for Tailoring Immunization Programs (HCD-TIP) provides “hands-on” tools and participatory approaches to working with communities to (1) understand reasons for low vaccine uptake among the un/undervaccinated and underserved populations, (2) co-design tailored solutions to address the identified challenge, and (3) implement and evaluate the solution with continued community engagement.^35^^,^^46^ This article discusses our use of ALPs to strengthen the capacity of PMs and HCWs at the state and LGA levels on HCD-TIP to improve data use for tailored immunization strategies, illustrated through the Strengthening Capacity for Immunization Data Use (SCID) pilot intervention in Northern Nigeria. We present policy recommendations to improve capacity-building interventions for immunization and other PHC interventions.

## METHODS

### Strengthening Capacity for Immunization Data Use Intervention

SCID is a capacity-building intervention that (1) used ALPs to train immunization PMs and HCWs on HCD-TIP iterative stepwise process for understanding and addressing barriers in immunization in partnership with communities and health facilities for tailored immunization programs; (2) provided templates to support the systematic use of data for diagnosis, intervention design, implementation, and evaluation; and (3) introduced incorporating the principle of “good enough,” which are critical and easy-to-take actions to be taken at each stage of the HCD-TIP process.^46^ This helps PMs and HCWs to generate simple, people-centered, culturally appropriate, feasible, and low-cost solutions to address the identified challenge.^46^ This aligns with the global Immunization Agenda 2030, which urges programs to prioritize “people-centered” immunization strategies for equitable coverage and systematically consider the needs of communities and the HCWs who serve them.^49^

### Theory of Change

**FIGURE 1:**
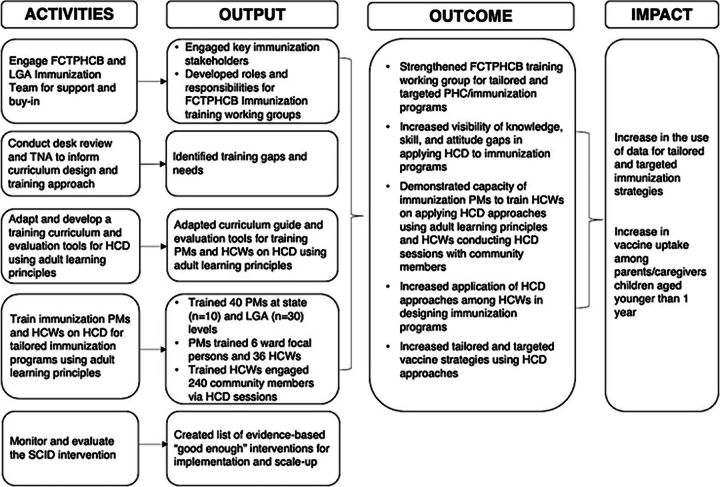
Strengthening Capacity for Immunization Data Use Intervention Theory of Change Abbreviations: FCTPHCB, Federal Capital Territory Primary Health Care Development Board; HCD, human-centered design; HCW, health care worker; LGA, local government area; PHC, primary health care; PM, program manager; TNA, training needs assessment; SCID, Strengthening Capacity for Immunization Data Use.

Our theory of change describes the hypothesized causal pathway between the intervention activities and their intended outputs and outcomes to improve the use of data for tailored immunization strategies (Figure 1). By incorporating ALPs in the TOT of state and LGA PMs on HCD-TIP approaches, they can efficiently cascade the training to HCWs, who will co-design tailored strategies with community members to address immunization barriers and improve vaccine uptake and coverage.

### Strengthening Capacity for Immunization Data Use Intervention Activities

The SCID intervention was implemented in the Federal Capital Territory (FCT), North-Central Nigeria, in collaboration with the FCT Primary Health Care Development Board (FCTPHCB). The intervention targeted 40 immunization PMs—10 state PMs and 30 LGA PMs, 42 HCWs—6 ward focal persons and 36 HCWs, and 240 community members (Box).

BOX Strengthening Capacity for Immunization Data Use Intervention Participants

**State program managers (PMs)** implement the national and state plan for the immunization program in the state and provide managerial, technical, and advisory support to the local government area (LGA) PMs. The 10 state PMs targeted for the Strengthening Capacity for Immunization Data (SCID) intervention included the following designations at the state level: state emergency routine immunization coordination center program manager, immunization officer, routine immunization desk officer, monitoring and evaluation officer, health educator, cold chain officer, disease notification and surveillance officer, director planning research and statistics, director primary health care, and members of the training working group. These individuals are members of the Federal Capital Territory Primary Health Care Development Board (FCTPHCB).

**LGA PMs** manage and provide supportive supervision of the immunization program at the ward and health facility levels. The SCID intervention targeted similar designations at the LGA levels, including the LGA director of primary health care, immunization officer, assistant immunization officer, routine immunization desk officer, cold chain officer, monitoring and evaluation officer, health educator, disease surveillance and notification officer, and community engagement focal persons. The 30 LGA PMs were identified and selected with the help of the FCTPHCB for the SCID intervention and data collection activities.

**Ward focal persons** (**WFPs)** are health care workers (HCWs) who supervise immunization services at the health facility level and collate facility-generated immunization data for submission to the LGA level. The 6 WFPs were identified and selected with the help of the FCTPHCB for the SCID intervention and data collection activities.

**HCWs** deliver immunization services to community members and record the number of children vaccinated and doses of vaccines administered in designated data tools. They comprise the health facility officer-in-charge, routine immunization (RI) focal person or vaccinator, and RI recorder. The 36 HCWs were identified and selected with the help of the FCTPHCB for the SCID intervention and data collection activities.

**Community members** are people who receive immunization services in their locality. The community members included in this intervention were community leaders, community secretaries/scribes, religious leaders, women leaders, youths, youth leaders, market women, and parents of (vaccinated and non-vaccinated) children aged younger than 2 years. These individuals were identified and selected for the SCID intervention through their community leaders with the help of the LGA PMs and HCWs.

Our approach to the intervention was participatory and targeted to the needs of the stakeholders at all levels. Four key activities were implemented between May and September 2023.

#### Stakeholder Engagement

**FIGURE 2:**
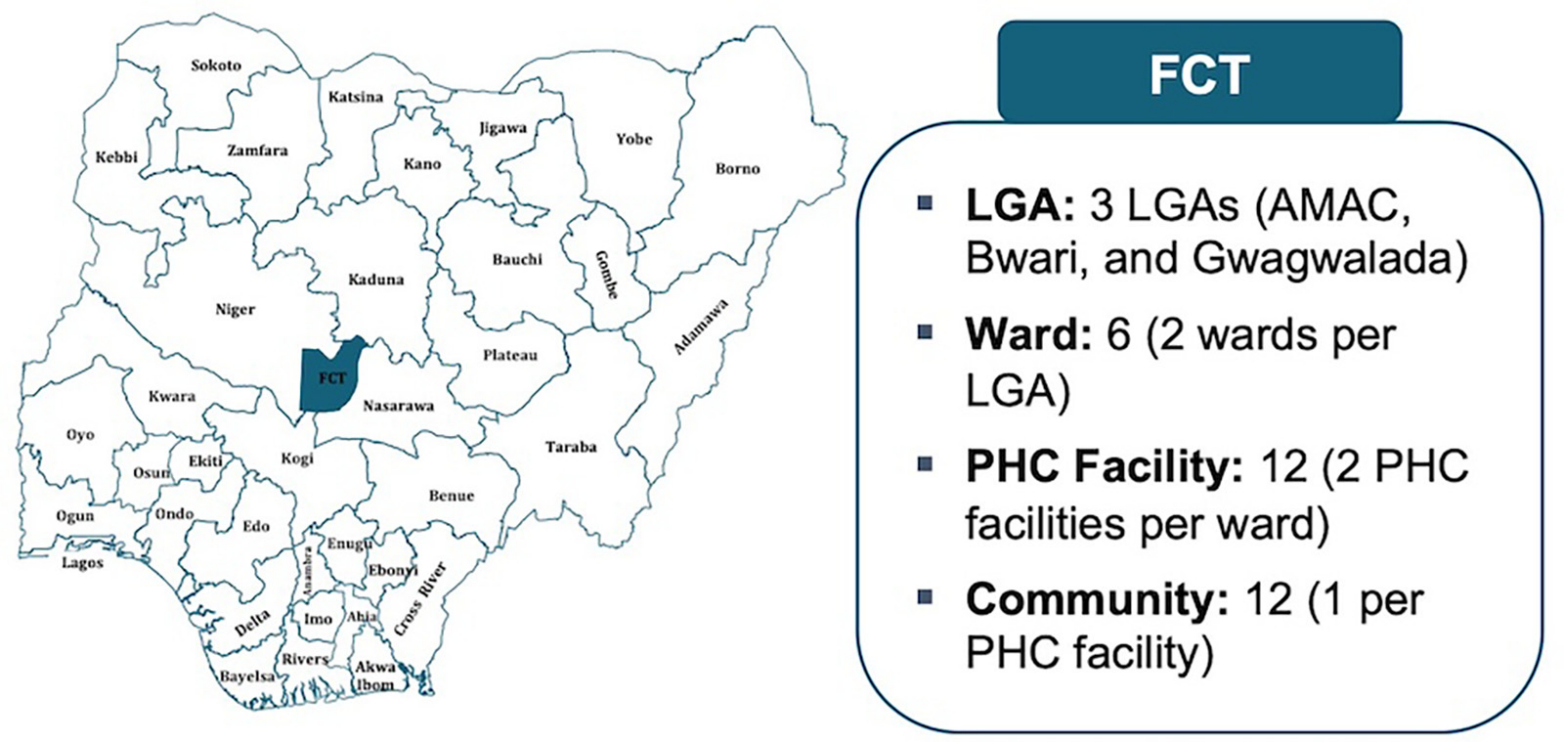
Strengthening Capacity for Immunization Data Use Intervention Study Sites Abbreviations: AMAC, Abuja Municipal Area Council; FCT, Federal Capital Territory; LGA, local government area; PHC, primary health care.

We engaged critical stakeholders at the FCTPHCB and LGA levels to sensitize them about the SCID intervention and garner their input and buy-in for the project. This engagement led to the purposive selection of the study sites based on coverages of the first and third doses of the pentavalent vaccine (Penta 1 and Penta 3) from the administrative data (i.e., DHIS2 and zero-dose proportion [high and low], demography [rural and urban], and accessibility [non-security-compromised areas]). One of the selected study sites, Abuja Municipal Area Council (AMAC), is among the prioritized zero-dose LGAs identified by NPHCDA for intensive intervention (Figure 2). The stakeholder engagement also led to the reactivation of the FCT Training Working Group (FCT-TWG) and the co-development of the FCT-TWG Standard Operating Procedure with the FCT-TWG.

Engaging key stakeholders at all levels is crucial to obtaining support for a realistic, scalable, and sustainable training program owned and driven by the government.

#### Desk Review and Training Needs Assessment

We conducted a desk review of existing literature to identify participatory and adult learning best practices in delivering immunization training and a training needs assessment (TNA) to identify the capacity gaps and needs on understanding, utilizing, and taking action based on immunization data, applying HCD approaches to immunization programs, and delivering immunization training. The TNA revealed (1) poor knowledge of dropout, zero-dose, and missed children; (2) competency gaps in data collection, analysis, interpretation, synthesis, and utilization to inform evidence-based strategies; (3) engagement with communities was mainly for awareness and mobilization activities and not to understand barriers to vaccination using local data and co-design interventions to improve vaccine uptake; (4) a need for training on data quality, analysis, and evidence-based strategies using local data; and (5) a preference for active participation, problem-solving, and site demonstration approaches in immunization training. Findings from the desk review and TNA were used to inform the training approach and curriculum design.

#### Curriculum Development and Template Adaptation

The study team, comprising experts in socio-behavioural science, HCD, immunization program, and ALPs, co-developed the curriculum and training guide for PMs and HCWs with the FCT-TWG in 2 phases in June 2023. Phase 1 was a 2-day curriculum and training guide development workshop; Phase 2 was a 5-day curriculum finalization activity. Based on the desk review and TNA findings, the curriculum integrated ALPs and technical and operational content to train PMs and HCWs on HCD-TIP. For example, the diagnosis phase of HCD-TIP included the ALPs of brainstorming using local data and case studies, the design phase incorporated the ALPs of group sessions, the implement phase comprised the ALPs of simulations/role play, and the evaluate phase added the ALPs of discussions (Table 1). For the technical content, we adapted the World Health Organization HCD-TIP training guide and template,^46^ the UNICEF HCD-TIP Workshop Deck,^46^ and harmonized recommendations from the FCT-TWG on training content and delivery approach for each of the gaps identified in the TNA. The operational content included a refresher on data sources, collection and management, indicators for assessing immunization performance and definition of zero-dose and missed children, data analysis and interpretation, and problem analysis and prioritization skills. ALPs of brainstorming and reflection exercises guided the curriculum development workshop.

**TABLE 1.** Summary of ALPs Applied During HCD-TIP Training of Immunization PMs and HWs in Nigeria

**HCD-TIP Stage**	**Learning Objectives**	**HCD-TIP Participatory Approaches and Tools**	**ALP**
Diagnose	Review and understand their local immunization data. Engage communities to agree on the priority group to reach. Use end user personas template to understand the BeSD of vaccination.	Gather relevant existing immunization data. Bring stakeholders together. Develop an end user persona using the HCD-TIP End User Persona Template containing BeSD questions. Collect new data to fill information gaps. Regroup to discuss findings and correct or validate any assumptions.	Group exercises and brainstorming sessions using case studies of local immunization data and context
Design	Transform insights from the diagnose phase into practical solutions.	Map the end user journey using the Journey to Health and Immunization Framework based on insights gathered during the diagnose stage, identify enablers and barriers along each step, and ensure a comprehensive understanding of the user experience. Identify the most significant barrier or areas of intervention using the Urgent vs. Important matrix as a decision-making tool. Develop a SMART objective. Generate ideas to address objective. Select an intervention idea using the Urgent vs Important matrix and create a prototype.	Reflections, discussions, and group exercises
Implement	Guide improvement by testing the prototype with a few end users. Develop an implementation plan. Select indicators to monitor and measure outputs and outcomes. Implement based on the agreed plan.	Conduct testing using the HCD-TIP Test Results Sheet. Develop an implementation plan using the HCD-TIP Implementation Planning Sheet. Discuss and select indicators to measure. Implement the intervention.	Brainstorming and simulations/role-play to act the solution implementation with some group members acting as the end users.
Evaluate	Monitor the progress of the implemented intervention. Input, review, and analyze indicator data. Identify and agree on improvements for subsequent monitoring cycles and processes to improve data quality. Use the HCD-TIP Evaluation Wrap-Up Sheet to document lessons learned.	Input and review updated indicator data on the HCD-TIP Implementation Monitoring Sheet comparing baseline and monitoring periods. Conduct stakeholder review of indicator data alongside the problem and design objective. Agree on improvements for the next cycle.	Group exercises and discussions mimicking the monthly data review meeting scenario and plenary feedback sessions

Abbreviations: ALP, adult learning principle; BeSD, behavioral and social drivers; HCD-TIP, human-centered design for tailoring immunization programs.

#### Training Using Adult Learning Principles

Our training delivery approach was guided by the participatory, experimental, and reflective ALPs and supported using the teach-back method for effective information delivery and reception.^50^ The study team and the FCT-TWG trained 10 state and 30 LGA PMs from the selected LGAs on the HCD-TIP steps, its templates, and the ALPs to apply, enabling them to effectively train HCWs (Table 1). Participants skills were built on how to “diagnose” immunization challenges with communities, turn insights or findings from the “diagnose” session into tailored and targeted prototype solutions (“Design”), “implement” the prototype solution, and monitor and evaluate the implemented intervention and document lessons learned (“Evaluate”). Participants used case studies developed by the FCT-TWG to aid group exercises and brainstorm along the steps of HCD-TIP. We also used posters, templates, and printed presentations to aid understanding. To address the gap in interpreting immunization data identified during the TNA, we had a hands-on session on navigating the DHIS2 platform, data visualization and interpretation, and best practices for using immunization data for decision-making.

We employed teach-back methods throughout the training. To ensure participants adequately received the information after each session, the facilitators asked them to repeat, present, and non-shamingly summarize what they understood, the key HCD-TIP components, how they would implement the activity, and the lessons learned. Facilitators’ presentations were followed routinely by questions and answers, and if participants misunderstood, the facilitators explained the information again, modifying the explanation to make it more straightforward.

The training delivery approach was guided by the participatory, experimental, and reflective ALPs and supported using the teach-back method for effective information delivery and reception.

Based on their participation and performance levels during the training, 3 state PMs and 12 LGA PMs (4 per LGA) were identified and selected to co-facilitate the training of HCWs as master trainers. These 15 PMs trained 42 HCWs (i.e., 6 ward focal persons and 36 HCWs) on the 4 HCD-TIP steps using participatory, experimental, and reflective ALPs supported with the teach-back method. Using their understanding of the communities they serve, their RI performance, and the skills they acquired, the HCWs created an initial HCD session plan. This plan included the zero-dose communities, timeline, and individuals to engage, including vulnerable and marginalized groups, for the first HCD sessions.

### Evaluation

We evaluated the effectiveness of the SCID intervention at 3 time periods using a mixed methods approach guided by the Kirkpatrick training evaluation model.^51^^,^^52^ Kirkpatrick’s model evaluates the performance of training programs along 4 levels: participant’s reaction/satisfaction with the training, participant’s knowledge and skills gained from the training (learning), participant’s behavior changes post-training, and results/outcomes obtained. Given our focus on assessing the training workshop’s effect on increasing the use of data for tailored immunization strategies and vaccination uptake among un/undervaccinated and underserved populations, we evaluated the 4 levels (Table 2).

**TABLE 2.** Summary of the Monitoring and Evaluation Method for SCID Intervention

**Intervention Phases**	**Kirkpatrick Evaluation Level and Description**	**Application to SCID Intervention**
Post-training	**Reaction:** To what degree do participants react favorably to the learning event?	The participant’s satisfaction with the SCID intervention using a self-administered survey.
Pre-and post-training	**Knowledge:** To what degree do participants acquire the intended knowledge, skills, and attitudes based on their participation in the learning event?	The knowledge and skills gained from participating in the SCID intervention and self-reported skills using a pre-and post-test survey.
Post-implementation (endline)	**Behavior:** To what degree do participants apply what they learned during training when they return to the job?	Whether the participant applied what was learned from the SCID training workshops in conducting HCD sessions with community members and co-designing targeted and tailored immunization strategies with communities using observation, surveys, and FGDs.
	**Results:** To what degree do targeted outcomes occur due to learning event(s) and subsequent reinforcement?	The outcome(s) from participating in the training using data from the surveys, DHIS2, IDIs, and FGDs.

Abbreviations: FGD, focus group discussions; HCD, human-centered design; IDI, in-depth interview; SCID, Strengthening Capacity for Immunization Data Use.

After the intervention, a survey was administered to conveniently sampled HCWs (36), LGA PMs (16), and state PMs (6) who were available for the assessment using a structured questionnaire. Seven focus group discussions (FGDs) were held with purposively selected state (1) and LGA (3) PMs and HCWs (3) using a semistructured interview guide. Observation was done during the HCD sessions with communities. Immunization rates were obtained from the DHIS2 between July 26 and August 24, 2023 (4 weeks), when the co-designed solutions were tested to assess any impact on immunization rates.

The differences between the pre-and post-training (quantitative data) were analyzed using Stata version 17 to estimate the effect of the intervention. The Wilcoxon signed rank and T-test were used to assess whether knowledge was statistically significant post-training. We also conducted other univariate descriptive statistics such as standard deviation and mean. The audio-recorded interviews were transcribed verbatim, coded, and analyzed thematically using Dedoose software. Exemplary statements were used for illustrative purposes. However, quotes were anonymized to protect respondents’ confidentiality and location.

### Ethical Approval

We received ethical approval from the FCT Health Research Ethics Committee (FHREC/2023/01/57/20-04-23). All study participants gave their informed oral consent before data collection.

## RESULTS

### Sociodemographic Characteristics of Training Participants

Pre- and post-training, we surveyed 80 participants, 41 HCWs, 28 LGA PMs, and 11 state PMs aged 25 to 59 years. Most females (85%) and males (79%) were HCWs and LGA PMs, respectively. Most respondents had been in their current roles for less than 6 years, LGA PMs (57%), HCWs (54%), and state PMs (46%). LGA PMs (29%) had more respondents who had occupied their roles for over 10 years.

### Participants’ Training Satisfaction and Quality

Using a post-training survey, we assessed participants’ reactions to the training, specifically satisfaction and quality. Thalheimer’s performance-focused surveys were adapted to evaluate the training quality because they gauge its success and produce actionable results to nudge action immediately.^53^^–^^55^ We also incorporated a survey question from Traicoff et al.^25^ The survey had participants respond to a series of questions to assess the expectations, relevance, delivery, training materials, and overall satisfaction using a Likert scale and statements to assess the training quality based on a rubric that ranked each statement as alarming, unacceptable, acceptable, and superior.

**FIGURE 3:**
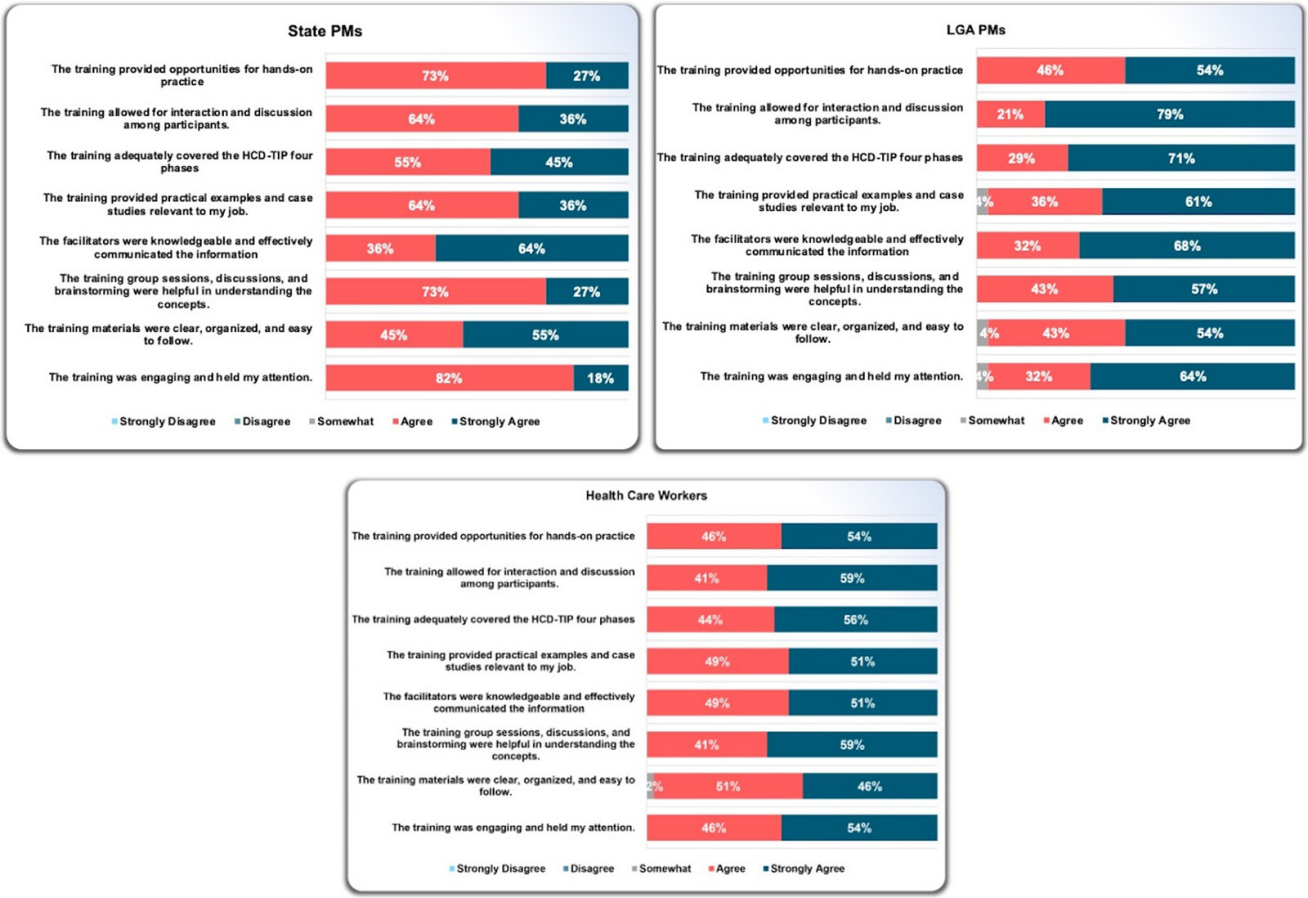
Participants' Reaction to the Strengthening Capacity for Immunization Data Use Training Delivery Abbreviations: HCD-TIP, human-centered design for tailored immunization program; LGA, local government area; PM, program manager.

None of the state PMs, LGA PMs, and HCWs felt that their training expectations were unmet. Instead, 18%–49% felt their training expectations were either “moderately,” “mostly,” or “completely” met. Specifically, 39%, 36%, and 32% of LGA PMs, state PMs, and HCWs, respectively, felt that their training expectations were “completely” met. Another 45%, 32%, and 20% of state PMs, LGA PMs, and HCWs, respectively, also said their training expectations were “mostly” met. Lastly, the training expectations of 49%, 29%, and 18% of HCWs, LGA PMs, and state PMs, respectively, were “moderately” met. In addition, the training was perceived as highly relevant to their current roles (ranging from 91% to 96%). All 3 groups of participants had high satisfaction levels in the training delivery (Figure 3). The most agreed or strongly agreed was that the training was engaging by state PMs (82%) and facilitated interaction through group sessions, discussions, and brainstorming by LGA PMs (79%) and HCWs (59%). All participants affirmed that the training materials adequately supported their learning experience of the HCD-TIP. State PMs (100%), HCWs (98%), and LGA PMs (96%) expressed a positive inclination to refer to the materials (guide, slides, and template/tools) later. Overall, there was a high level of satisfaction with the training workshop among LGA PMs (100%), state PMs (91%), and HCWs (85%).

We assessed participants’ reactions to the training quality across 9 domains. Participants were not exposed to the rubric ranking standards. Most participants’ responses fell within the rubric of “acceptable” and “superior” (45%–100%) confidence levels in applying the concepts learned during the training to their daily job tasks. For responses that fell into “alarming” and “unacceptable,” further investigation was done through the identification number on the survey form, and support was provided to clarify all concerns. The Supplement presents participants’ responses to each statement and rubric ranking.

For future HCD-TIP training, to ensure training satisfaction and quality are met, participants, through an open-ended question, recommended providing continued guidance and mentorship in using the HCD-TIP approaches, using simplified HCD-TIP templates for each group (PMs and HCWs), training more PMs and HCWs, extending training days, and offering residential training for immersive and distraction-free training environments.

The training content and the participatory delivery approach met the participants’ expectations, were relevant to their job, and motivated them to apply and prioritize what they had learned in their daily job tasks.

### Participants’ Learning Results

We used technical exercises and teach-back methods during the training and pre-and post-tests to assess the knowledge and skills gained in PMs and HCWs. The technical exercises that involved group exercises used local immunization data and case studies, allowing PMs and HCWs to practice new skills and learning. At the same time, the facilitators provided feedback during the exercises. The teach-back method employed throughout the training enabled us to evaluate their training delivery skills and learning and gave participants constructive feedback. Our daily facilitator’s meetings allowed us to debrief on the day’s performance and address any gaps identified.

The Wilcoxon signed-rank test was used to compare the PMs’ pre-and post-training evaluation scores and assess whether knowledge changes were statistically significant post-training. The pre-and post-test mean scores showed a statistically significant improvement in the knowledge and competence of the PMs by 21% in the post-training evaluation (*P*<.001). We used the 2-sample t-test to compare evaluation scores pre- and post-training by a cadre of PMs and participants’ years in current roles. By cadre of PMs, pre-training, both groups had similar knowledge and competence levels (*P*=.8212). However, there was a significant difference in post-training evaluation scores between state and LGA PMs (*P*=.0056). Additionally, PMs’ years of experience in their current roles did not significantly influence their pre-training (*P*=.6887) and post-training evaluation (*P*=.9879) (Table 3).

**TABLE 3.** Knowledge and Competence Scores Pre- and Post-Evaluation Among PMs

	**Pre-training**		**Post-training**	
	**Mean Score±SD**	***P* Value**	**Mean Score±SD**	***P* Value**
All PMs (N=39)	17.54±4.51	<.001	21.18±4.77	<.001
**Cadre**				
State PMs (n=11)	17.27±3.41	.8212	24.45±3.33	.0056
LGA PMs (n=28)	17.64±4.93		19.69±4.60	
**Years in current role**				
>6 years (n=18)	16.67±4.73	.6887	21.17±4.71	.9879
<6 years (n=21)	18.29±4.30		21.19±4.95	

Abbreviations: LGA, local government area; PM, program manager, SD, standard deviation.

We used the paired t-test to compare the HCWs’ pre- and post-training evaluation scores. There was a statistically significant (*P*<.001) increase in knowledge and competence scores from pre-training (mean of 9.97) to post-training (mean of 15.88). We also assessed the difference in scores by the cadre of HCWs using the paired t-test and HCWs’ years in current roles using a 2-sample t-test. The training had a significant and positive impact on the knowledge and competence of the officer-in-charge (*P*=.0246), recorder (*P*=.0003), and RI focal person HCWs (*P*=.0004). In pre-training, we found that the number of years in the current role had no significant impact on the pre-training evaluation scores of HCWs (*P*=.7613). Similarly, the post-training scores showed no statistically significant difference in mean scores between HCWs’ years in their current role (*P*=.6106) (Table 4).

**TABLE 4.** Knowledge and Competence Scores Pre- and Post-Evaluation Among HCWs

	**Pre-training**		**Post-training**	
	**Mean Score±SD**	***P* Value**	**Mean Score±SD**	***P* Value**
All HCWs (N=41)	9.97±3.76	<.001	15.88±4.34	<.001
**Cadre**				
Officer-in-charge (n=12)	11.50±4.58	.0246	16.17±4.20	.0246
Recorder (n=10)	9.3±4.35	<.001	16.8±3.26	<.001
RI focal person (n=14)	8.5±4.82	<.001	15.0±4.26	<.001
WFP (n=5)	11.8±4.44	.1749	15.8±2.28	.1749
**Years in current role**				
>6 years (n=18)	9.72±5.10	.7613	16.22±3.87	.6106
<6 years (n=23)	10.17±4.36		15.61±3.74	

Abbreviations: HCW, health care worker; LGA, local government area, SD, standard deviation; WFP, ward focal person.

### Participants’ Behavioral Changes

We assessed the behavioral changes of the trained participants at the endline, specifically whether they applied what was learned from the SCID workshops in effectively conducting HCD sessions with community members and co-designing targeted immunization programs to improve vaccine uptake using observation, survey, and FGDs. The survey respondents were primarily females (61%), with ages ranging from 24 to 59 years. HCWs had the lowest average age of 39.50±6.66 years, while LGA PMs had the highest average age of 46.92±9.12 years. State PMs (67%), LGA PMs (56%), and HCWs (53%) had been in their current roles for less than 6 years.

Between July 24 and August 27, 2023, the study team, FCT-TWG members, and state and LGA PMs observed the 42 trained HCWs use their immunization data and HCD-TIP approaches and tools to effectively conduct 2 distinct HCD sessions in 12 communities with 240 community members (120 community members per HCD session), including vulnerable groups and missed communities and co-design 24 targeted and tailored prototype solutions for testing (Table 5). Given the tight project timeline, only the 12 prototypes developed during the first HCD session were tested for 4 weeks, and lessons learned were documented for improvement.

**TABLE 5.** Examples of Co-designed “Good Enough” Strategies With Community Members

**Facility (Community)**	**Who Are the Unimmunized?**	**Prioritized Barriers**	**Design Objective**	**Prototype Solution**
Hulumi PHC (Brazil)	Mothers of children aged younger than 1 year	No transport fare to visit Hulumi PHC for vaccination	For mothers of children aged younger than 1 year to change from delaying vaccination to timely vaccination by addressing no transport fare to visit Hulumi PHC for vaccination.	Operation bring vaccination to the community
Kutunku PHC (Angua Hausawa/Hayi Kabir)	Hausa caregivers of the Hayi-Kabir community	Inability of Hausa women in the community to know/remember their immunization dates due to illiteracy	For hesitant and complacent caregivers of Hayi Kabir community to change to seeking vaccination for their children by addressing the forgetfulness of the immunization schedule.	TUNAWA: Appointment of reminder champions among the women as an integrated component of immunization campaigns
Chikakore PHC (Chikakore)	Indigenes (30%) and non-indigenes (70%)	The poor health facility orientation and packaging	For indigenes and non-indigenes of the Chikakore community to change to accessing services by addressing poor health facility packaging and orientation.	Repackaging Chikakore PHC
Jigo PHC (Jigo)	Some members from Pambara	Forgetfulness of caregivers on the immunization schedule	For members of the Pambara and Jigo communities to change from missing their immunization dates to coming to the facility on the right schedule by addressing reminder issues and the number of immunization days.	Starting reminders using the tickler box before the immunization date and creating more immunization days in a week
Ushafa PHC (Ushafa)	Settlers of Chappe Ruga	Fear of AEFIs	For fathers in Chappe Ruga to change from vaccine hesitancy due to AEFIs to accepting vaccines by addressing the lack of knowledge about AEFI management and the importance of immunization.	Dialogue with the community leader of Chaape Ruga to create a platform for health workers to educate fathers and mothers in the Chappe community on AEFIs.
Township Clinic (Angwan Madaki)	Full housewives	Fear of AEFI and lack of husband's permission for immunization	For the unimmunized Hausawa caregivers to change from vaccine hesitancy to vaccine acceptance by addressing the fear of AEFIs and the lack of husband permission.	Community dialogue with men and fathers of the community.
Dagiri PHC (Dagiri)	Fulani settlement households	Misconceptions and misinformation about vaccines and infertility	For hesitant husbands to change to being receptive to vaccines by addressing the cultural misconception about vaccines and infertility.	Engagement of community Imam to sensitize followers on vaccine safety, debunking misinformation on infertility.
	Igbo woman and other residents	Fear of AEFIs and lack of knowledge of AEFI management	For the vaccine-hesitant residents of Dagiri to change from hesitancy to acceptance by addressing their fear of AEFIs.	Vaccine hesitancy mapping during campaigns, monthly follow-up with community leaders, and leveraging community ceremonies for 10-minute immunization/AEFIs talk.

Abbreviations: AEFI, adverse events following immunization; PHC, primary health care.

Three months post-training, most LGA PMs (75%), HCWs (72%), and state PMs (67%) reported an improved understanding of HCD approaches due to the training. LGA PMs (75%) and HCWs (61%) strongly agreed to have applied the HCD-TIP principles in designing tailored immunization programs using data, compared to state PMs (67%) who indicated “agree.” The qualitative findings validated these findings, where LGA PMs and HCWs revealed that the HCD-TIP training enhanced their skills and capacity in diagnosing and designing tailored solutions.

Three months post-training, 75% of LGA PMs, 72% of HCWs, and 67% of state PMs reported an improved understanding of HCD approaches due to the training.

*Based on the diagnosis thing, we were able to identify that some of our mothers really want to come out for immunization, but due to some reasons like their husband doesn’t allow them to come, fear of adverse events following immunization (AEFI). Then, we went further to design it with the community and how to get this problem solved. Then, during the implementation that was when we met with the community leaders and the husbands of the mothers. The breastfeeding mothers were called just to tell them how to manage AEFI at home, and we told the husbands the importance of immunization so it would enable them to let their wives come out for immunization. Then, during the evaluation, it was when we went back for community outreach to check whether what we went there for was really listened to.* —HCW

Most LGA PMs (81%), HCWs (75%), and state PMs (50%) strongly agreed that the workshop positively influenced their approach to immunization programs by enhancing collaboration with communities, communication skills, and data-driven approaches. During the qualitative interviews, participants also reported a change in work practice following the HCD-TIP training workshop, especially in their data management skills and effectively engaging communities to generate insights on behavioral and social drivers of vaccine uptake.

*It has really improved me because we normally find it difficult to track them down to the communities, but with this information, we are able to go into the community to actually know the core problem of the issue and from there, some certain intervention through the knowledge we have gathered was put it in place. —*LGA PM

### Outcome

We assessed the reported outcomes from the training using surveys, FGDs, and data from the DHIS2. The survey and FGD assessed the perceived impact of the training on service delivery and immunization uptake. From the DHIS2, we obtained data on RI performance (Penta 1 and Penta 3) post-HCD sessions across the 12 communities where the prototypes of the first HCD session were tested for 4 weeks between July 26 and August 24, 2023.

**FIGURE 4:**
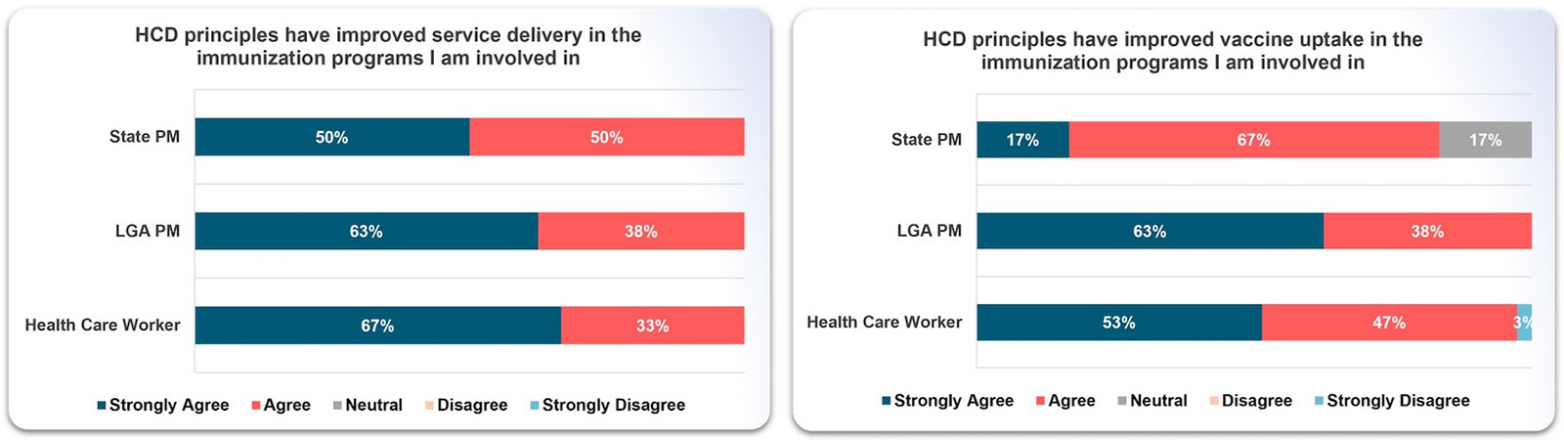
Perceived Impact of the Training on Service Delivery and Vaccine Uptake Among PMs and Health Care Workers Abbreviations: HCD, human-centered design; LGA, local government area; PM, program manager.

Most state PMs, LGA PMs, and HCWs either strongly agreed or agreed that applying HCD principles impacted service delivery and vaccine uptake in the 12 participating health facilities (Figure 4).

**FIGURE 5:**
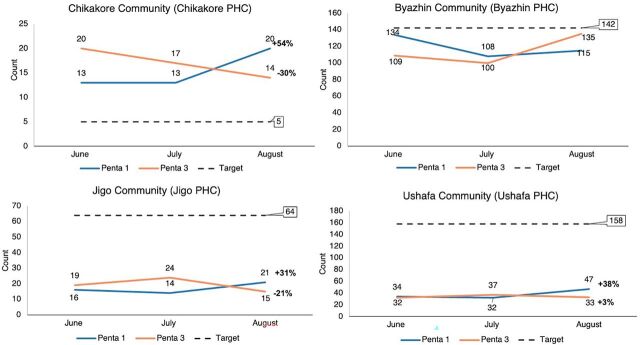
Number of Vaccine-Age Children Who Received Penta 1 and Penta 3 Vaccines During the Test Period in Bwari LGA Abbreviations: LGA, local government area; Penta 1, first dose of pentavalent vaccine; Penta 3, third dose of pentavalent vaccine; PHC, primary health care.

**FIGURE 6:**
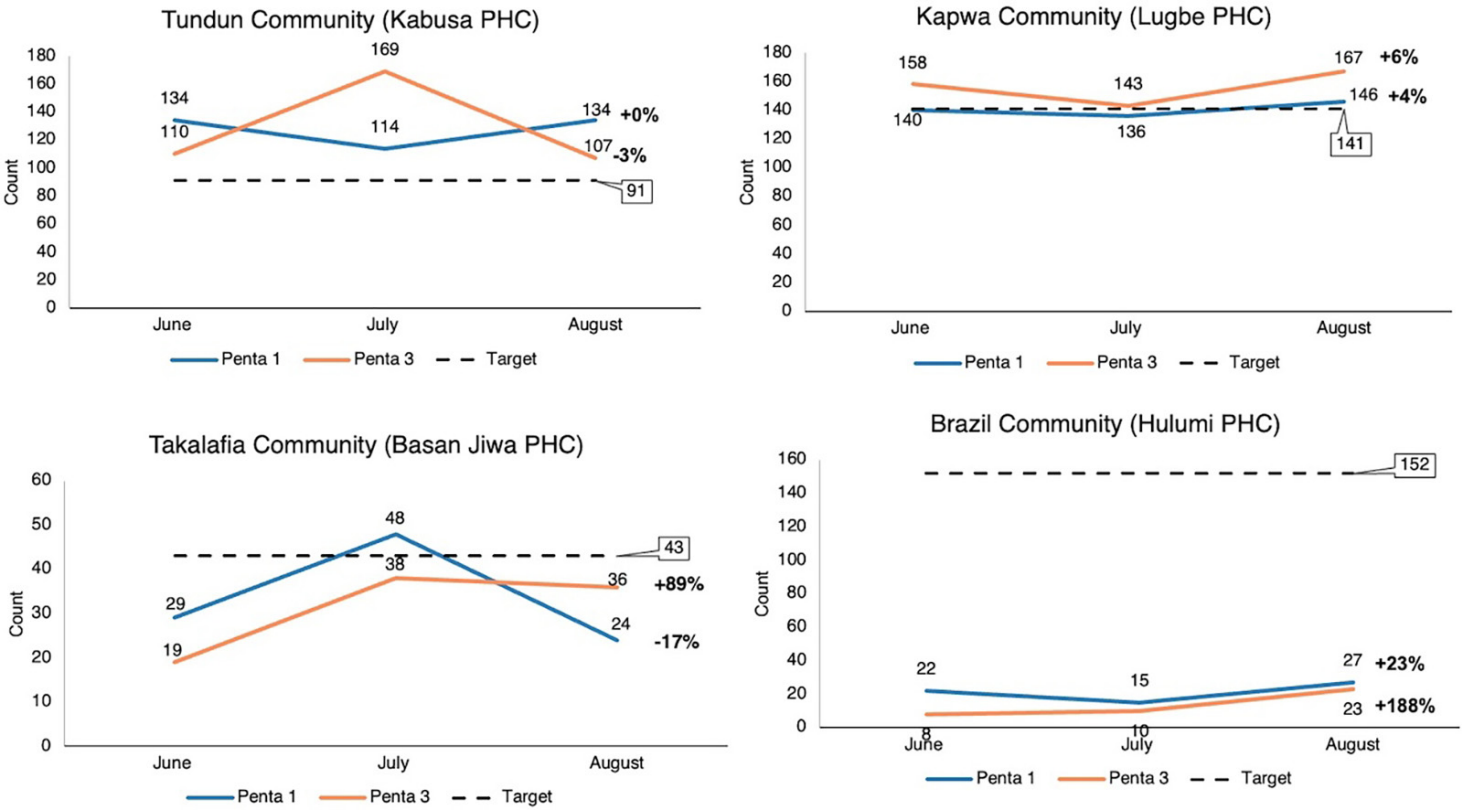
Number of Vaccine-Age Children Who Received Penta 1 and Penta 3 Vaccines During the Test Period in AMAC LGA Abbreviations: AMAC, Abuja Municipal Area Council; LGA, local government area; Penta 1, first dose of pentavalent vaccine; Penta 3, third dose of pentavalent vaccine; PHC, primary health care.

**FIGURE 7:**
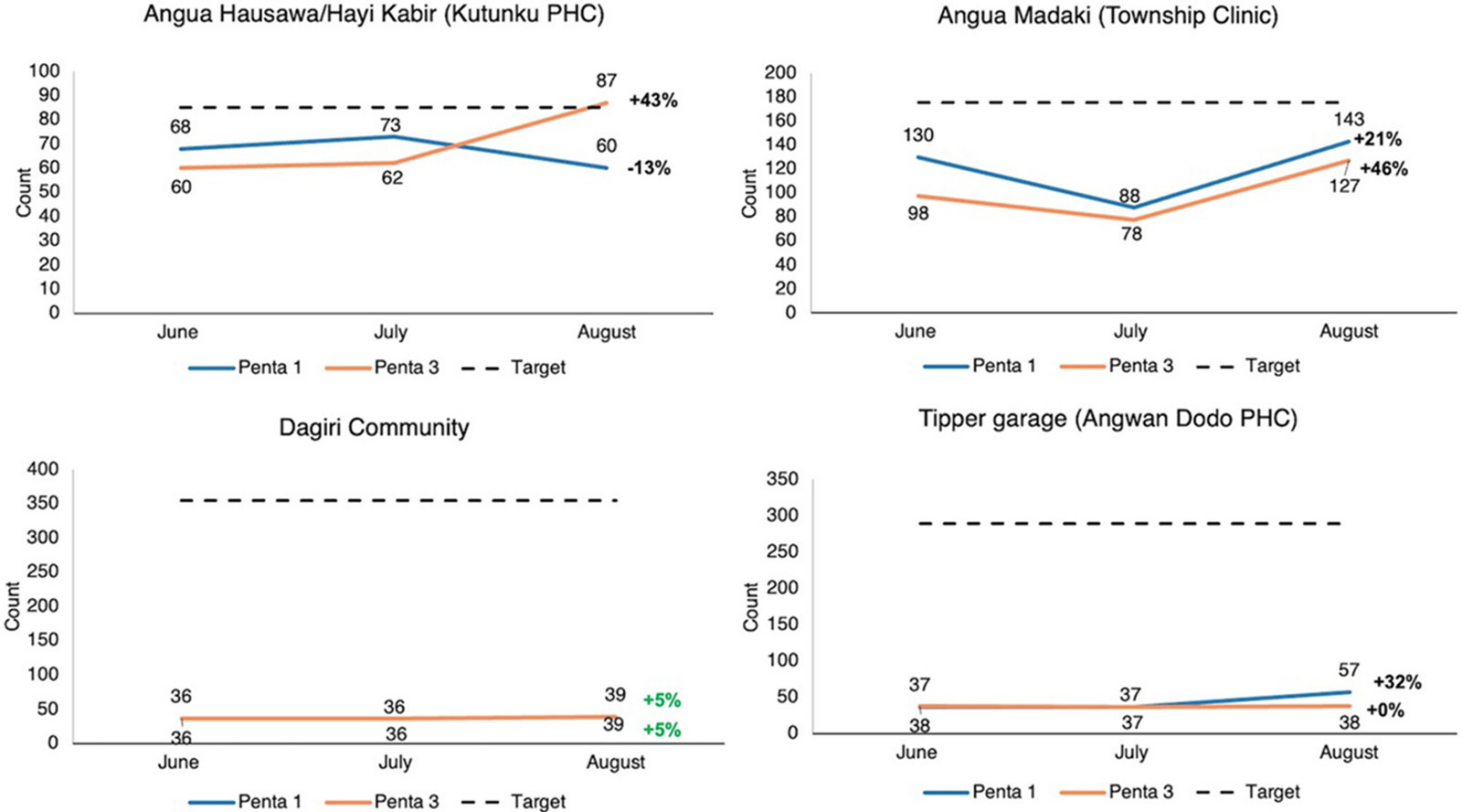
Number of Vaccine-Age Children Who Received Penta 1 and Penta 3 Vaccines During the Test Period in Gwagwalada LGA Abbreviations: LGA, local government area; Penta 1, first dose of pentavalent vaccine; Penta 3, third dose of pentavalent vaccine; PHC, primary health care.

The DHIS2 records showed varied improvements in Penta 1 and Penta 3 uptake across the 12 communities assessed from the 3 LGAs. The Chikakore Community in Bwari LGA recorded the most improvement for Penta 1 (54%) (Figure 5), and the Brazil Community (Hulumi PHC) in AMAC LGA (Figure 6) and Angwa Madaki (Township Clinic) (Figure 7) had the most improvement for Penta 3 uptake (188%) and (46%), respectively. PMs and HCWs confirmed this during the interviews.

*In Angwa Madaki township, you’ll see in July, their Penta 3 was 78 and in August, 127; you could see that it’s magic; and if you can also see Byazhim community, Penta-3 in July was 100 and August 135. So, this is evidence-based data that we can say yes, it’s yielding results.* —State PM

Participants reported challenges in implementing the HCD-TIP principles, such as conflicting activities, identifying solutions and prototypes, shortage of workforce, and the lengthy volume of the HCD-TIP templates. Despite these challenges, the trained participants leveraged existing structures, such as the monthly ward development committee meetings, to apply the HCD in their immunization program. The training manual and guides also served as reference material to facilitate using the HCD approaches in their immunization program. Participants also mentioned that technical support and funding are the key drivers in applying the HCD-TIP techniques.

Participants reported challenges in implementing the HCD-TIP principles, such as conflicting activities, identifying solutions and prototypes, shortage of workforce, and the lengthy volume of the HCD-TIP templates.

## DISCUSSION

The SCID intervention successfully used ALPs to design and deliver TOT for immunization PMs and HCWs on HCD-TIP approaches to improve data use for tailored immunization programs and equitable immunization coverage.

None of the participants (state PMs, LGA PMs, and HCWs) felt their training expectations were unmet. However, the degrees to which expectations were met vary. Participants had expected simplified data tools (e.g., the HCD-TIP templates) to ease the data documentation burden, extended training days, and residential training for immersive and distraction-free training environments. Although these expectations were unmet, most participants (91%–96%) perceived the training as highly relevant to their current roles. State PMs (82%), LGA PMs (79%), and HCWs (59%) agreed or mostly agreed to be satisfied with the training content and delivery approach because it used local immunization data and context as case studies, was interactive, and employed participatory approaches. These varied satisfaction levels could be attributed to their roles, experiences, and expectations. For example, state PMs occupy strategic and leadership positions that expose them to numerous training and workshops, making them more satisfied with interactive and engaging training methods. LGA PMs are involved in program implementation; thus, group sessions, discussions, and brainstorming align well with their needs for practical and actionable insights. They may also value collaboration and shared problem-solving, explaining their high satisfaction with the interactive nature of the training. In contrast, HCWs, who are involved with service delivery and face competing activities and heavy workloads, may prefer practical and hands-on demonstrations, which could affect their overall satisfaction. Other studies have also reported heterogeneity in training groups’ expectations and satisfaction in meeting the needs of all participants.^56^^,^^57^ We also found an increased confidence in applying learnings taught across the tiers assessed. This is consistent with other studies that used case studies and incorporated participatory approaches during training, which improved the learning experience, motivated learners, and increased confidence to apply the knowledge and skills learned.^25^^,^^58^^–^^61^ A participatory approach that may have also contributed to the satisfaction observed in our study could be that the training content was co-developed with the users relevant to their needs and the program. This is because adults are most interested in learning subjects if it has immediate relevance to their work and can help solve real-life problems.^28^ This calls for program implementers to conduct TNAs in the design phase to ensure the training content and delivery are tailored to the learner’s needs and expectations.

We found a statistically significant improvement in the knowledge and competence of the PMs and HCWs after training (*P*<.001). This outcome is consistent with other studies that applied ALPs to various health programs, including childhood vaccination.^25^^,^^30^^,^^58^^,^^62^^–^^66^ Similar to our finding, critical contributors to the reported increase in knowledge and competence levels included the training materials, self-directed learning, brainstorming and discussion exercises, simulations, and practice exercises,^25^^,^^30^^,^^58^^,^^62^^,^^63^ resulting in participation of the proposed intervention.^67^ Additionally, follow-up or on-the-job mentoring is required to confirm knowledge retention and application in work practices.^58^ This can be done using existing platforms, such as supportive supervision activities or data review meetings, to strengthen capacity and promote peer learning on tailored immunization programs to improve vaccine uptake.^23^^,^^27^^,^^28^^,^^68^^–^^70^

Evidence exists on other capacity-building models used in immunization programs to increase knowledge and competencies,^31^^,^^71^ such as mentoring, on-the-job training, and audit-focused supervision^72^; whole-staff training and train-the-trainer approaches^73^; short, targeted training methods^31^; peer-mentoring and Whatsapp^74^; immunization curriculum^31^; and using community-based participatory approach.^75^ However, integrating ALPs in TOT presents a unique opportunity for capacity-building, as it focuses on designing and delivering immunization training to the needs of the learners in a collaborative and participatory manner, even in low-resource settings.^24^^,^^25^^,^^28^^,^^76^

Our study demonstrated that training on HCD-TIP using ALPs positively influenced behavioral changes in work practices among PMs and HCWs, especially in managing data, engaging communities to generate insights on behavioral and social drivers of vaccine acceptance, and co-designing data-driven and tailored strategies. This is consistent with other studies that applied ALPs in immunization and other health program training, resulting in improved attitudes, data management, communication skills with caregivers, and the creation of defaulter lists.^30^^,^^70^^,^^77^ The introduction of the new vaccines provides an opportunity to strengthen the skills of HCWs through training.^78^^,^^79^ Integrating training on HCD-TIP into existing immunization training programs can consistently enhance their skills and reach more people.

Our study demonstrated that training on HCD-TIP using ALPs positively influenced behavioral changes in work practices among PMs and HCWs.

Additionally, our trained PMs efficiently trained HCWs on applying HCD approaches for targeted immunization strategies using ALPs. These HCWs then used their health facility immunization data and HCD-TIP approaches and templates to engage with community members in identifying reasons for non- or undervaccination, co-design 24 “good enough” prototype solutions, and document outputs from community engagement activities.

The participatory approach to training these HCWs, followed by the hands-on application of the knowledge gained in conducting HCD sessions with communities during the monthly ward development committee meetings, provision of tools, and supportive supervision by state and LGA PMs during the HCD sessions, facilitated the use of HCD approaches for tailored strategies. These strategies have been shown to strengthen the capacity of the health workforce and the RI system.^72^^,^^80^^,^^81^ Leveraging existing community engagement activities to diagnose and co-design solutions with communities could address challenges with conflicting activities and further strengthen their skills and familiarity with the HCD-TIP templates. Also, continuous capacity-building or peer-learning platforms, such as the data review meetings, are needed to encourage PMs and HCWs to use HCD-TIP approaches in zero-dose and missed communities.

Furthermore, while the timeline was short to assess long-term impact, Penta 1 and Penta 3 vaccines uptake increased across the 12 participating communities and health facilities during the testing of the co-designed tailored strategies. The training approach, complemented by other targeted interventions for zero-dose prioritized LGAs, such as the Optimized Integrated RI Session,^82^^,^^83^ may have contributed to increased vaccine uptake, especially in AMAC LGA, where 1 of the participating communities and health facilities recorded increased Penta 3 coverage. The over 100% coverage reported for Penta 3 in 1 of the communities could be attributed to target population issues due to faulty denominators peculiar in Nigeria^84^^,^^85^ and other African countries.^86^ This calls for innovations to address denominator issues and increase the accuracy of immunization program targets.^13^ Other contextual factors that may have influenced the intervention and outcomes included using the existing supportive supervision structure at the FCTPHCB, where the trained PMs provided HCWs with ongoing mentoring and supervision as they conducted HCD sessions at the community level. Also, considering Nigeria’s top-down management culture, securing the FCTPHCB’s buy-in influenced the state and LGA PMs and HCWs to participate in the training. However, the reliance on self-directed learning without guidance for implementation and evaluation has been seen as a potential unintended consequence of adult learning in training programs, which may lead to inconsistencies in the quality and effectiveness of training.^87^ Suggestions for training programs include actively involving learners in the learning process, tailoring the training to the learners’ specific needs and interests, and continuously evaluating training programs.^30^^,^^87^ Tools, such as Thalheimer’s survey^54^ and Kirkpatrick’s training evaluation model,^52^ can be used to assess the training program’s effectiveness and identify improvement areas for refinement.

Global advisory bodies and scholars have called on countries to enhance the use of existing data for tailored immunization programs to achieve equitable vaccine coverage.^13^^,^^88^ HCD approaches have proven effective in understanding barriers to vaccine uptake, co-creating tailored and locally relevant solutions with the target population, and improving immunization uptake and coverage in marginalized communities.^34^^,^^37^^,^^44^^,^^48^^,^^89^^,^^90^ PMs and HCWs should be encouraged to integrate the HCD-TIP approaches while engaging with community members and offering nonmonetary incentives for those who actively use the HCD-TIP approaches and tools to diagnose and design tailored strategies to improve vaccine uptake. Additionally, integrating training interventions into existing structures, such as the FCTPHB, and building the capacity of the FCT-TWG members on HCD-TIP to drive the coordination and design of capacity-building interventions and facilitate sessions ensures scalability and sustainability.

HCD approaches have proven effective in understanding barriers to vaccine uptake and co-creating tailored and locally relevant solutions with the target population.

While the HCD approach is flexible, the core principles of HCD-TIP recommend pilot testing with a smaller population ahead of implementation planning.^46^ Our co-designed prototypes from the first HCD session with communities were tested within 4 weeks, providing room for documenting lessons learned and improvement. Consistent with our approach, several studies pilot-tested their co-designed prototypes for 6 months,^91^^–^^93^ and 2 weeks,^94^ while other approaches are recommended for estimating the effectiveness of prototype solutions.^95^^,^^96^ Long-term studies are needed to test the effectiveness of the tested “good enough” strategies in improving service delivery and coverage.

### Implications

Based on the observations and study findings, training programs must be optimized to enhance their effectiveness and impact on using data for tailored strategies through the following efforts.

- Conducting a TNA to understand the specific knowledge gaps and challenges among participants and to tailor the training content and delivery to address those pressing needs.
- Co-developing the training content with learners or users to ensure that it addresses their needs and expectations and is relevant to the program.
- Adopting an adult learning approach in delivering capacity-building interventions to improve knowledge and competence.
- Integrating training interventions into existing government structures for ownership and sustainability.
- Continuous evaluation of the training program during and after training to identify areas of improvement for refinement.

Training programs must be optimized to enhance their effectiveness and impact on using data for tailored strategies through the following efforts.

HCD-TIP requires continued community engagement.^46^ Although HCWs engage with community members, continuous capacity-building, a platform for peer learning, and mentorship are needed to promote HCD-TIP approaches during community engagement activities. Countries can use adult learning methods like data review meetings to promote peer learning and mentoring on HCD-TIP approaches and strengthen capacity for data reporting and quality.^24^^,^^28^^,^^29^^,^^68^^–^^70^ Lastly, policies must incorporate behavioral science in immunization data and programs to better understand behavioral and social drivers of vaccine acceptance and hesitancy for targeted interventions.

### Limitations

Given the limited project time and budget constraints, only a subset of wards and PHC facilities within the selected LGAs were included in the study. While this limits the generalization of our study findings to other PMs and HCWs in Nigeria, they can still be applied to settings and immunization workforces with similar contexts. In addition, the survey questionnaire that assessed participants’ knowledge and skills, training satisfaction and quality, and behavioral changes were self-reported and subject to social desirability bias. We compensated for this by using a mixed methods approach, allowing us to understand the context of the reported data. Although RI data review meetings are meant to be conducted monthly at the LGA level, logistical and financial challenges hindered the conduct of these meetings during the study period, thus limiting our ability to address data reporting and quality issues and the opportunity for peer learning. To mitigate this, we conducted a post-intervention evaluation and data validation meeting with HCWs and PMs in each intervention LGA. These meetings allowed us to identify and address data reporting and quality issues and exchange knowledge and experiences. Furthermore, the limited project period did not allow us to assess the impact of the 2 HCD sessions on immunization coverage and tests of the solutions generated during the second HCD session. Although our plan was not to assess long-term impact, we tested the prototype solutions from the first HCD sessions and documented changes in service provision, immunization rates, and lessons learned valuable for program implementers, policymakers, and donors to strengthen the immunization training program and improve equitable vaccine coverage in Nigeria.

## CONCLUSION

Incorporating participatory approaches such as the ALPs in TOT for training PMs and HCWs improves their knowledge and skills in effectively engaging the communities and co-designing tailored interventions to improve vaccination uptake. Policymakers should consider adopting a holistic approach that focuses on building the capacity of PMs and HCWs using the ALPs, actively engaging communities using HCD-TIP approaches and tools, using data for tailored interventions, and applying behavioral science principles in immunization programs to improve vaccine uptake.

## Supplementary Material

GHSP-D-23-00465_supplement.pdf

## Notes

**Peer Reviewed**

**First Published Online:** September 18, 2024.
