# Supplementary material for: Strengthening Capacity for Tailored Immunization Programs Using Adult Learning Principles: A Case Study from Nigeria
Source: Glob Health Sci Pract. 2024 Oct 29;12(5):e2300465. doi: 10.9745/GHSP-D-23-00465 (PMC11521550; doi:10.9745/GHSP-D-23-00465)
Supplement: GHSP-D-23-00465_supplement.pdf [file GHSP-D-23-00465_supplement.pdf]

Supplement to: Obi-Jeff C, Oguntimehin F, Adejumo A, et al. Strengthening capacity for tailored immunization programs using the adult learning principles: a case study from Nigeria. *Glob Health Sci Pract*. 2024;12(5):e2300465. https://doi.org/10.9745/GHSP-D-23-00465

SUPPLEMENT. Participants' reactions to the immunization training quality

Domain	Statement	Rubric Ranking Standard	%		
			State PMs	LGA PMs	HWs
**On-the-job performance**	HOW ABLE ARE YOU to put what you've learned into practice in your work? **CHOOSE THE ONE OPTION** that best describes your current readiness.				
	My CURRENT ROLE DOES NOT ENABLE me to use what I learned.	Alarming	0	0	0
	I AM STILL UNCLEAR about what to do and/or why to do it.	Alarming	0	0	0
	I NEED MORE GUIDANCE before I know how to use what I learned.	Unacceptable	0	4	12
	I NEED MORE EXPERIENCE to be good at using what I learned.	Acceptable	18	25	22
	I CAN BE SUCCESSFUL NOW in using what I learned (even without more guidance or experience).	Superior	73	64	56
	I CAN PERFORM NOW AT AN EXPERT LEVEL in using what I learned.	Superior/Over-confident	9	7	2
**Learner comprehension**	Now that you've completed the HCD-TIP training, how well do you feel you understand the concepts taught? **CHOOSE ONE.**				
	I am still at least SOMEWHAT CONFUSED about the concepts.	Alarming	0	0	0
	I am now SOMEWHAT FAMILIAR WITH the concepts.	Unacceptable	9	7	15
	I have a SOLID UNDERSTANDING of the concepts.	Acceptable	45	36	10
	I AM FULLY READY TO USE the concepts in my work.	Superior	45	54	66
	I have an EXPERT-LEVEL ABILITY to use the concepts.	Superior/Over-confident	0	4	2
**Technical content confidence**	Select the **one best answer** that best describes what the training will enable you to do if anything.				
	It DID NOT enable me to UNDERSTAND NEW CONCEPTS or USE NEW SKILLS.	Unacceptable	0	0	0
	It will enable me to UNDERSTAND SOME NEW CONCEPTS but did NOT PREPARE ME TO USE NEW SKILLS on the job.	Unacceptable	9	0	5
	It will enable me to BEGIN TRYING NEW SKILLS on the job.	Acceptable	27	18	24
	It will enable me to CONFIDENTLY USE NEW SKILLS on the job.	Superior	45	39	32

Supplement to: Obi-Jeff C, Oguntimehin F, Adejumo A, et al. Strengthening capacity for tailored immunization programs using the adult learning principles: a case study from Nigeria. *Glob Health Sci Pract*. 2024;12(5):e2300465. https://doi.org/10.9745/GHSP-D-23-00465

<table>
<tr><th rowspan="2">Domain</th><th rowspan="2">Statement</th><th rowspan="2">Rubric Ranking Standard</th><th colspan="3">%</th></tr>
<tr><th>State PMs</th><th>LGA PMs</th><th>HWs</th></tr>
<tr><td></td><td>It will enable me to BE THOROUGHLY CONFIDENT AND PRACTICE IN USING NEW SKILLS on the job.</td><td>Superior/Over-confident</td><td>18</td><td>32</td><td>34</td></tr>
<tr><td rowspan="6">Learner motivation to apply</td><td colspan="5">Regarding the concepts taught in the workshop, how motivated WILL YOU BE to USE these skills in your work? Select the one best answer.</td></tr>
<tr><td>I WILL NOT MAKE THIS A PRIORITY when I get back to my job.</td><td>Unacceptable</td><td>0</td><td>0</td><td>0</td></tr>
<tr><td>I will make this a PRIORITY-BUT A LOW PRIORITY-when I get back to my job.</td><td>Unacceptable</td><td>0</td><td>0</td><td>2</td></tr>
<tr><td>I will make this a MODERATE PRIORITY when I get back to my job.</td><td>Acceptable</td><td>18</td><td>11</td><td>17</td></tr>
<tr><td>I will make this a HIGH PRIORITY when I get back to my job.</td><td>Acceptable</td><td>73</td><td>61</td><td>51</td></tr>
<tr><td>I will make this one of my HIGHEST PRIORITIES when I get back to my job</td><td>Superior</td><td>9</td><td>25</td><td>27</td></tr>
<tr><td rowspan="7">Opportunities to practice</td><td colspan="5">Which of the following were true about the opportunities you were given to practice? Select all that apply.</td></tr>
<tr><td>I was given ALMOST NO PRACTICE.</td><td>Unacceptable</td><td rowspan="3">0</td><td rowspan="3">7</td><td rowspan="3">12</td></tr>
<tr><td>I was given INADEQUATE AMOUNTS OF PRACTICE.</td><td>Unacceptable</td></tr>
<tr><td>I DID NOT GET ENOUGH HELPFUL FEEDBACK WHEN WE WERE PRACTICING.</td><td>Unacceptable</td></tr>
<tr><td>I was given TOO MUCH PRACTICE.</td><td>Alarming</td><td>0</td><td>4</td><td>7</td></tr>
<tr><td>I was OFTEN ASKED TO PRACTICE SOMETHING RIGHT AFTER WE LEARNED IT.</td><td>Acceptable</td><td rowspan="2">100</td><td rowspan="2">82</td><td rowspan="2">29</td></tr>
<tr><td>I generally RECEIVED SUFFICIENT AND HELPFUL FEEDBACK AFTER WE PRACTICED A TASK.</td><td>Acceptable</td></tr>
<tr><td rowspan="6">Instructor(s) Performance</td><td colspan="5">Which of the following were true about the training facilitators? Select all that apply.</td></tr>
<tr><td>Generally DID A GOOD JOB in facilitating the training.</td><td>Superior</td><td>45</td><td>64</td><td>63</td></tr>
<tr><td>Too often HURRIED through the content in a SUPERFICIAL manner.</td><td>Unacceptable</td><td rowspan="2">0</td><td rowspan="2">0</td><td rowspan="2">0</td></tr>
<tr><td>Was OFTEN UNCLEAR or DISORGANIZED.</td><td>Unacceptable</td></tr>
<tr><td>Demonstrated DEEP SUBJECT-MATTER KNOWLEDGE.</td><td>Acceptable</td><td rowspan="2">55</td><td rowspan="2">29</td><td rowspan="2">22</td></tr>
<tr><td>Showed HIGH LEVELS OF REAL-WORLD EXPERIENCE relevant to the topic.</td><td>Acceptable</td></tr>
</table>

Supplement to: Obi-Jeff C, Oguntimehin F, Adejumo A, et al. Strengthening capacity for tailored immunization programs using the adult learning principles: a case study from Nigeria. *Glob Health Sci Pract*. 2024;12(5):e2300465. https://doi.org/10.9745/GHSP-D-23-00465

<table>
<tr><th rowspan="2">Domain</th><th rowspan="2">Statement</th><th rowspan="2">Rubric Ranking Standard</th><th colspan="3">%</th></tr>
<tr><th>State PMs</th><th>LGA PMs</th><th>HWs</th></tr>
<tr><td></td><td>MOTIVATED ME TO ENGAGE ACTIVELY IN THE LEARNING.</td><td>Acceptable</td><td></td><td></td><td></td></tr>
<tr><td rowspan="6">Perceived relevance</td><td colspan="5">From your perspective, how valuable are the concepts taught in the workshop? HOW MUCH WILL THEY HELP YOU IMPROVE YOUR IMMUNIZATION COVERAGE OUTCOMES?</td></tr>
<tr><td>Will NOT HELP ME to improve my outcomes.</td><td>Unacceptable</td><td>0</td><td>0</td><td>0</td></tr>
<tr><td>Will HELP ME SLIGHTLY to improve my work outcomes.</td><td>Unacceptable</td><td>0</td><td>0</td><td>12</td></tr>
<tr><td>Will HELP ME TO A MODERATE AMOUNT to improve my work outcomes.</td><td>Acceptable</td><td>18</td><td>11</td><td>15</td></tr>
<tr><td>Will HELP ME SIGNIFICANTLY to improve my work outcomes.</td><td>Superior</td><td>82</td><td>82</td><td>68</td></tr>
<tr><td>Concepts taught ARE NOT RELEVANT to my work.</td><td>Alarming</td><td>0</td><td>0</td><td>0</td></tr>
<tr><td rowspan="6">After-learning support</td><td colspan="5">After the training, when you begin to apply your new knowledge in your role, which of the following supports are likely to be in place for you? Select all that are likely to be true</td></tr>
<tr><td>MY MANAGER/SUPERVISOR/SUPERIOR WILL ACTIVELY SUPPORT ME with key supports like time, resources, advice, and/or encouragement.</td><td>Superior</td><td>36</td><td>39</td><td>49</td></tr>
<tr><td>I will approach a MENTOR OR COLLEAGUE to guide me in applying the learning to my work.</td><td>Acceptable</td><td rowspan="3">55</td><td rowspan="3">50</td><td rowspan="3">49</td></tr>
<tr><td>I will regularly receive support from THE FACILITATORS or the TRAINING WORKING GROUP to help me in applying the learning to my work.</td><td>Acceptable</td></tr>
<tr><td>I will use the TRAINING TOOLS like the Facilitators Guide, Training Manual, HCID-TIP Templates, or reference materials to guide me in applying the learning to my work.</td><td>Acceptable</td></tr>
<tr><td>I feel I will NOT get much direct support but will rely on my own initiative.</td><td>Alarming</td><td>0</td><td>0</td><td>0</td></tr>
<tr><td rowspan="3">Perceived effectiveness of the learning experience</td><td colspan="5">If someone asked you about the effectiveness of the learning experience, would you recommend the learning to them? Select only ONE</td></tr>
<tr><td>The learning was TOO INEFFECTIVE to recommend.</td><td>Alarming</td><td>0</td><td>4</td><td>2</td></tr>
<tr><td>The learning was INEFFECTIVE ENOUGH THAT I WOULD BE HESITANT to recommend it.</td><td>Alarming</td><td>0</td><td>7</td><td>10</td></tr>
</table>

Supplement to: Obi-Jeff C, Oguntimehin F, Adejumo A, et al. Strengthening capacity for tailored immunization programs using the adult learning principles: a case study from Nigeria. *Glob Health Sci Pract*. 2024;12(5):e2300465. https://doi.org/10.9745/GHSP-D-23-00465

**Domain**	**Statement**	**Rubric Ranking Standard**	**%**		
			**State PMs**	**LGA PMs**	**HWs**
	The learning was NOT FULLY EFFECTIVE, BUT I would recommend it IF IMPROVEMENTS WERE MADE to the learning.	Unacceptable	0	0	0
	The learning was NOT FULLY EFFECTIVE, BUT I would still recommend it EVEN IF NO CHANGES WERE MADE to the learning.	Acceptable	0	0	5
	The learning was EFFECTIVE, SO I WOULD RECOMMEND IT.	Acceptable	55	54	32
	The learning was VERY EFFECTIVE, SO I WOULD HIGHLY RECOMMEND IT.	Superior	45	32	44
